# Predicting Obsessive-Compulsive Disorder Events in Children and Adolescents in the Wild Using a Wearable Biosensor (Wrist Angel): Protocol for the Analysis Plan of a Nonrandomized Pilot Study

**DOI:** 10.2196/48571

**Published:** 2023-11-14

**Authors:** Kristoffer Vinther Olesen, Nicole Nadine Lønfeldt, Sneha Das, Anne Katrine Pagsberg, Line Katrine Harder Clemmensen

**Affiliations:** 1 Applied Mathematics and Computer Science Technical University of Denmark Kongens Lyngby Denmark; 2 Child and Adolescent Mental Health Center Copenhagen University Hospital Mental Health Services Copenhagen Hellerup Denmark; 3 Department of Clinical Medicine Faculty of Health and Medical Sciences University of Copenhagen Copenhagen Denmark

**Keywords:** machine learning, obsessive-compulsive disorder, children, teens, adolescents, OCD, AI, artificial intelligence, mental health, Empatica E4, prediction, physiological signal, mental health care, wearable, monitoring, obsessive compulsive disorder

## Abstract

**Background:**

Physiological signals such as heart rate and electrodermal activity can provide insight into an individual’s mental state, which are invaluable information for mental health care. Using recordings of physiological signals from wearable devices in the wild can facilitate objective monitoring of symptom severity and evaluation of treatment progress.

**Objective:**

We designed a study to evaluate the feasibility of predicting obsessive-compulsive disorder (OCD) events from physiological signals recorded using wrist-worn devices in the wild. Here, we present an analysis plan for the study to document our a priori hypotheses and increase the robustness of the findings of our planned study.

**Methods:**

In total, 18 children and adolescents aged between 8 and 16 years were included in this study. Nine outpatients with an OCD diagnosis were recruited from a child and adolescent mental health center. Nine youths without a psychiatric diagnosis were recruited from the catchment area. Patients completed a clinical interview to assess OCD severity, types of OCD, and number of OCD symptoms in the clinic. Participants wore a biosensor on their wrist for up to 8 weeks in their everyday lives. Patients were asked to press an event tag button on the biosensor when they were stressed by OCD symptoms. Participants without a psychiatric diagnosis were asked to press this button whenever they felt really scared. Before and after the 8-week observation period, participants wore the biosensor under controlled conditions of rest and stress in the clinic. Features are extracted from 4 different physiological signals within sliding windows to predict the distress event logged by participants during data collection. We will test the prediction models within participants across time and multiple participants. Model selection and estimation using 2-layer cross-validation are outlined for both scenarios.

**Results:**

Participants were included between December 2021 and December 2022. Participants included 10 female and 8 male participants with an even sex distribution between groups. Patients were aged between 10 and 16 years, and adolescents without a psychiatric diagnosis were between the ages of 8 and 16 years. Most patients had moderate to moderate to severe OCD, except for 1 patient with mild OCD.

**Conclusions:**

The strength of the planned study is the investigation of predictions of OCD events in the wild. Major challenges of the study are the inherent noise of in-the-wild data and the lack of contextual knowledge associated with the recorded signals. This preregistered analysis plan discusses in detail how we plan to address these challenges and may help reduce interpretation bias of the upcoming results. If the obtained results from this study are promising, we will be closer to automated detection of OCD events outside of clinical experiments. This is an important tool for the assessment and treatment of OCD in youth.

**Trial Registration:**

ClinicalTrials.gov NCT05064527; https://clinicaltrials.gov/study/NCT05064527

**International Registered Report Identifier (IRRID):**

DERR1-10.2196/48571

## Introduction

Increased heart rate and electrodermal activity (EDA) are associated with mental phenomena such as fear, anxiety, anger, contamination disgust, embarrassment but also happiness [1]. Decreased heart rate and increased EDA are associated with sadness and mutilation-related disgust [1]. These negative emotions are seen in obsessive-compulsive disorder (OCD). Several types of OCD symptoms are linked with specific emotional profiles (eg, fear of contamination and contamination disgust, fear of harming or violating others, and guilt and shame), which could be detected using affective computing. Thierfelder et al [2] showed that an increase in heart rate and a decrease in heart rate variability (HRV) are indicative of an increase in stress during OCD events. Heart rate and HRV show distinctive patterns of anxiety when patients are exposed to fearful situations and relief when performing compulsive behavior. These patterns were found to be highly different between individuals but useful for distinguishing between cognitive stress, exposure and response prevention (ERP) episodes, and physical activity.

OCD is a burdensome psychiatric disorder that affects up to 3% of youths (individuals younger than 18 years) [3-5]. Obsessions are repetitive and intrusive thoughts that cause negative emotions such as anxiety, disgust, embarrassment, shame, guilt, and feelings of incompleteness, that is, marked distress [6]. Compulsions are repetitive or ritualized behaviors or actions the patient feels compelled to perform, despite their sometimes nonsensical nature [7]. Distress can arise from performing compulsions and when prevented from performing compulsions.

First-line treatment for OCD is cognitive behavioral therapy with ERP [8,9]. In the context of OCD treatment, ERP refers to approaching OCD symptom–triggering stimuli (exposure) and refraining from performing rituals and compulsions (response prevention). During ERP, patients are asked to intermittently report their level of distress [10]. This information helps the therapist adjust the interventions. Awareness of distress trajectories within and across ERP sessions is also a tool used to increase therapeutic learning [11,12]. Therefore, monitoring distress levels during exposure is important for optimizing ERP and measuring the severity of symptoms in OCD. Similarly, when collected over time, distress can provide information about the progression and improvement of the condition [13].

Many symptoms of OCD are directly related to specific objects or situations that cannot be realistically reproduced in clinical settings. Automatic, continuous detection of these OCD signals would facilitate close monitoring of patient treatment progress and allow for more targeted interventions and evaluation of treatment in the real world [14,15]. Ideally, distress would be objectively and affordably assessed in a noninvasive manner.

Recently, machine learning methods applied to data collected using smartwatches have demonstrated success in classifying stress levels [16,17], recognizing emotions [18,19], detecting panic attacks [20], and detecting hand washing—a common compulsion in individuals with OCD [15]. Thierfelder et al [2] demonstrated that recordings of heart rate and HRV using wearable devices may also be used to identify distress in patients with OCD caused by exposure during ERP sessions. Additionally, relief through compulsive behavior is reflected in the recorded heart rate and HRV, but the results also suggest that a change in the environment or simple distractions may reduce anxiety.

This paper aims to assess the possibility of using machine learning models for the detection of OCD events in the wild based on signals from wrist-worn devices. Automatic detection of OCD events will allow targeted interventions in the real world to improve OCD treatment. To the best of our knowledge, this is the first study of this kind. We aim to investigate the relationship between personalized and generalized predictive models by comparing the predictive performance of models trained on each participant individually and models trained on a sample of participants. In this paper, we also weigh our methodological decisions. Our research questions may be summarized as follows: (1) Is it feasible to collect enough data (physiological signals and tags) from individual participants to evaluate the feasibility of developing models? (2) To what extent do participants adhere to wearing the biosensor? (3) Is the number of event tags associated with OCD severity? (4) Do features extracted from physiological signals during OCD episodes in the wild resemble features during controlled ERP sessions in the clinic? (5) Can OCD episodes in the wild be predicted using physiological signals from a wearable biosensor? and (6) To what extent do predictive models generalize to newly recorded data from the same subjects or new subjects?

## Methods

### Overview

A complete description of the study design, recruitment, and data collection procedures are reported in the Wrist Angel study protocol, which was submitted for publication prior to the collection of data from the last participant [21].

### Participants

**Figure 1:**
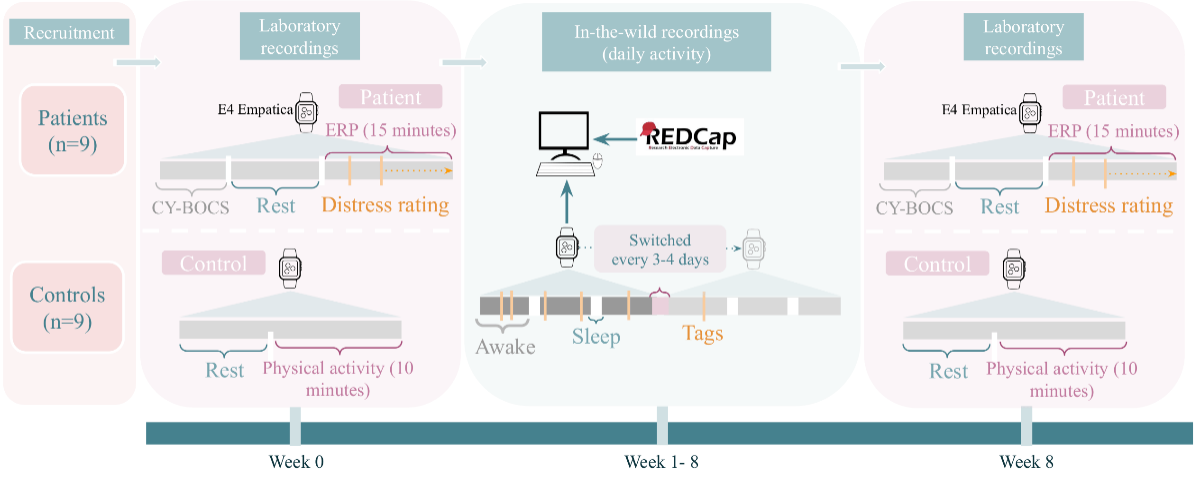
Design of the study and data collection procedure. Before and after an 8-week data collection period, all participants take part in a clinical recording session in the lab. Participants with an OCD diagnosis complete a clinical interview that assesses OCD severity and an ERP session. Participants with no psychiatric diagnosis engage in physical activity. During the in-the-wild recordings, participants wear the Empatica E4 biosensor daily during waking hours for 8 weeks and are asked to tag events of distress related to their OCD symptoms for patients, whereas nonclincal participants tag events of intense fear. Researchers meet with participants approximately every 3 days to switch wristbands. The collected biosignal data is then transferred to the hospital server. CY-BOCS: Children’s Yale-Brown Obsessive Compulsive Scale; ERP: exposure and response prevention; OCD: obsessive-compulsive disorder; REDCap: Research Electronic Data Capture.

A total of 18 youths aged 8 to 16 years and their parents were included in this study, and data were collected for 8 weeks. Nine participants had an OCD diagnosis (patients) and 9 youths presented without a psychiatric diagnosis (controls). Other studies have predicted aggressive behavior from wearables worn by 20 youths in 87 hours (10 consecutive days) or behavioral synchrony between father and their child with Down syndrome from wearables worn by 12 families in a 7-minute free play [22,23]. Although the number of participants is low, we followed each participant for 8 weeks, and we argue it will be enough for a feasibility study of prediction possibilities (as a minimum within individuals). Additionally, we are targeting a high emotional response, facilitating a stronger correlation between biosignals and emotional response. We present a brief description of the data acquisition illustrated in Figure 1 using the E4 wristband (Empatica) [24].

### Ethical Considerations

This study was approved by the ethics committee of the Capital Region of Denmark on June 17, 2021 (H-18010607-79689). Participants received written and verbal information about the study and the plan for disseminating results before they or their legal guardians signed the consent forms. We were not aware of any major risks or benefits for participants associated with participation. Adverse reactions to the biosensor throughout the study were monitored.

### Laboratory Recordings

Before (week 0) and after (week 8) data acquisition, each participant partook in laboratory experiments to obtain recordings of physiological signals in a controlled environment. Physiological signals were collected using the Empatica E4 wristband under rest and stressful conditions. During the rest period, participants were asked to sit still and quietly, without talking or touching each other, and to relax to the best of their ability. To simulate stressful conditions, control participants engaged in physical activity for approximately 10 minutes, while patients completed an ERP exercise. During the ERP task, patients approach OCD symptom–provoking stimuli and refrain from compulsions for up to 15 minutes led by a mental health professional in the presence of their participating parent. Patients provided subjective units of distress, that is, they rated their level of distress on a scale from 0 (no distress) to 10 (extreme distress), every few minutes.

### In-the-Wild Recordings

The youth and 1 of their parents were asked to wear the Empatica E4 biosensor daily during waking hours for 8 weeks. Patients were asked to press the event tag button when they felt stressed by their OCD symptoms. Parents of patients were asked to tag every time they noticed their child was distressed by their OCD symptoms. Control youth were asked to push the button when they felt very scared, and control parents were asked to push the button when they noticed that their child felt very scared. Researchers met with participants up to twice a week during the 8 weeks to exchange biosensors full of data with empty fully charged biosensors.

### Measures

#### Physiological Signals

The E4 wristband contains a photoplethysmographic (PPG) sensor, an EDA sensor, a 3-axis accelerometer, an optical thermometer, and an event tag button that records a point in time when pressed. PPG uses infrared light to measure changes in blood volume, that is, blood volume pulse (BVP). BVP is sampled 64 times per second and can be used to calculate the timings of the interbeat interval (IBI) and the heart rate [25]. Under conditions of slight movement (<30% of the time), it is possible to use the IBI to compute HRV. However, this constraint is often too rigid and results in the removal of entire signals, making this approach impractical for robust data acquisition in the wild. The EDA sensor measures the skin conductance sampled 4 times per second. The accelerometer measures movement on 3 axes at a sampling rate of 32 times per second. The optical thermometer measures skin temperature at a sampling rate of 4 times per second.

#### Clinical Measures

Participants were assessed with the Children’s Yale-Brown Obsessive Compulsive Scale (CY-BOCS) [26] before the start of the observation period. The CY-BOCS is the gold standard measure of OCD symptom severity [26]. Ten items (five items each for obsessions and compulsions) are summed to a total severity score [26]: (1) How much time is spent on obsessions and compulsions? (2) To what extent do obsessions and compulsions interfere with normal functioning? (3) How much distress do obsessions and compulsions (or not performing compulsions) cause? (4) To which extent are you able to resist obsessions and compulsions? and (5) To which extent do you have control over obsessions and compulsions? Five obsession or compulsion items can be summed to obtain obsession or compulsion severity scores, respectively. The CY-BOCS severity score has demonstrated high internal consistency and convergent and discriminant validity [10].

The CY-BOCS also includes a checklist for common symptoms, which are presented in 15 categories. These include seven obsession categories: (1) contamination, (2) aggressive, (3) sexual, (4) superstitious, (5) religious or moral, (6) hoarding, and (7) miscellaneous, and the eight categories for compulsions include (1) cleaning or washing, (2) checking, (3) repeating, (4) counting, (5) hoarding, (6) superstitious behaviors, (7) involving others in rituals, and (8) miscellaneous. Meta-analysis supports the existence of a 4-factor structure of OCD symptoms representing the following factors: symmetry, forbidden thoughts, cleaning, and hoarding [27]. Symmetry includes thoughts about symmetry and counting, ordering, and repeating compulsions. Forbidden thoughts include thoughts of an aggressive, sexual, and religious nature. The cleaning category covers contamination, somatic obsessions, and cleaning behaviors. Finally, the hoarding factor includes obsessions about losing or saving things and saving compulsions. We will accumulate the number of endorsed items belonging to each category to produce a category score as previously done in factor structure studies [28].

### Analysis Plan

#### Overview

This work will focus on the in-the-wild recordings obtained from the youth participants only. We will treat marked OCD events as binary targets and design predictive models of these targets. We will limit the data to physiological signals and marked events from youth and only consider data recorded in-the-wild during the 8 weeks.

#### Assessing the Feasibility of In-The-Wild OCD Event Prediction

Continuous data acquisition as described previously presents several practical issues and barriers whose effects must be evaluated for wide-scale use. Even though the Empatica E4 wristband is generally considered a nonintrusive device, some patients might consider it a conspicuous piece of hardware and fear strangers might judge them on a potentially sensitive problem. Additionally, participants might need to adjust to wearing the bracelet and learn to tag events at the appropriate time, not forget to tag events, or even wear the wristband over time.

We qualitatively evaluate the potential issues and barriers based on feedback and comments from participants and their parents during data collection approximately every 3 days. We associate the number of days the wristband has been worn; the number of recorded hours and tagged events per day as a function of time to the CY-BOCS severity and type, number; and the severity of OCD symptoms from the preobservation period clinical interviews.

#### Preprocessing of Signals and Feature Extraction

We examine the length and end time of each recording session to verify that participants only wear the Empatica E4 wristband during waking hours. In the case where participants wear the wristband during sleep, we intend to manually exclude these recordings.

We extract features from the heart rate, skin temperature, EDA, and BVP signals recorded by the Empatica E4 wristband. The preprocessing of the signals is outlined as follows:

1. The skin temperature is processed using a sixth-order Butterworth low-pass filter with a cutoff frequency of 1 Hz.
2. The heart rate is extracted using the PPG sensor in the Empatica E4 wristband using a proprietary Empatica algorithm. The recordings of the heart rate are used as is and require no further preprocessing.
3. As discussed previously, the E4 wristband only provides the IBI signal in periods of low movement. Therefore, this signal is highly discontinuous and may not be useful for extracting the HRV during short time windows. Instead, the recorded BVP signal is segmented using a rolling window of 5 seconds with a time step of 1 second [29]. For each segment, we determine the noise level based on the skew and kurtosis [30]. If the kurtosis is less than *εk*=–0.5 and the magnitude of the skew is less than *ɛs*=1, the segment is deemed a low-noise segment and included in the segmenting process. Features are extracted from each selected segment and then averaged to obtain the final features.
4. Features extracted from the EDA signal have previously been found to be good predictors of stress [31,32]. However, the EDA signal is also affected by physical activity and environmental factors, leading to expected participant and temporal variations unrelated to the stress level. The EDA signal is first processed using a sixth-order Butterworth low-pass filter with a cutoff frequency of 1 Hz. The signal is then normalized to the interval [0,1] to reduce the effects of subject and temporal variations. The normalized signal was decomposed into its tonic and phasic parts using the *NeuroKit2* library [33] for Python (Python Software Foundation). The tonic part is a slowly varying baseline conductivity, and the phasic part is a series of event-related high-frequency peaks. The unnormalized EDA signal is retained for feature extraction as the normalization may inadvertently remove explanatory features related to the level of the signal.

**Figure 2:**
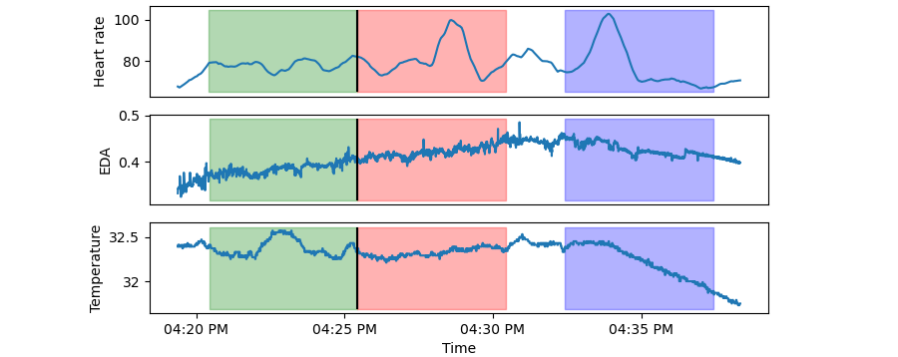
Illustration of the windowing strategy around each tagged OCD event (black). Positive cases are sampled from the period leading up to the tagged event (green) and a buffer period of 5 minutes is applied after the event from which no observations can be sampled (red). Negative cases are sampled randomly from the remaining time periods (blue). EDA: electrodermal activity.

For feature extraction, we apply a window strategy around each tagged OCD event, as shown in Figure 2. We use the 5-minute period before each tag as positive observations. The 5 minutes after each tag is removed is a buffer period to avoid event contamination in nonevent windows. Nonevent windows of equal temporal length are sampled randomly from the remaining time periods as negative observations. By increasing the number of randomly sampled negative observations, we investigate the performance differences between models trained on class-balanced data sets and models trained using more but imbalanced data.

Within each extracted window, we compute the features tabulated in Table 1.

1. We compute the following time domain features of each used signal: mean (SD), minimum, maximum, and gradient of a least square regression. For the BVP signal, we also compute the minimum, maximum, and average of the slope fitted in each segment. For the heart rate and EDA signal where the level is expected to carry some explanative power, we also compute the quartiles and IQR.
2. From the BVP signal, we detect systolic peaks using the *NeuroKit2* library [33] within each low-noise segment. These peaks are used to calculate the interbeat interval and the successive differences of this interval. We then compute the final features as the average of the IBIs and the root mean square of the successive differences calculated in all low-noise segments.
3. For the phasic component of the EDA signal, we compute the number of peaks as well as the average peak amplitude and response time.
4. For the EDA signal, we compute the power of the frequency bands: ultralow frequency (0.01-0.04 Hz), low frequency (0.04-0.15 Hz), high frequency (0.15-0.4 Hz), and ultrahigh frequency (0.4-1.0 Hz). These frequency bands are computed for the unnormalized EDA signal and the phasic component.
5. For the BVP signal, we compute the mean (SD), median, IQR, minimum, maximum, and sum of the frequency content. These values are further separated into real and imaginary parts.

**Table 1:** Features extracted from each used signal.

Feature	Heart rate	Temperature	EDA^a^	BVP^b^
Mean and SD	µ_HR_, σ_HR_	µ_TEMP_, σ_TEMP_	µ_sig_, σ_sig_ sig ∈ {EDA, Phasic}	µ_BVP_, σ_BVP_
Median	p^50^_HR_	—^c^	p^50^_sig_ sig ∈ {EDA, Phasic}	p^50^_BVP_
25th and 75th quartiles	p^25^_HR_, p^75^_HR_	—	p^25^_sig_, p^75^_sig_ sig ∈ {EDA, Phasic}	—
IQR	p^75^_HR_-p^25^_HR_	—	p^75^_sig_ – p^25^_sig_ sig ∈ {EDA, Phasic}	—
Minimum and maximum	min_HR_, max_HR_	min_TEMP_, max_TEMP_	min_sig_, max_sig_ sig ∈ {EDA, Phasic}	min_BVP_, max_BVP_
Slope	δ_HR_	δ_TEMP_	δ_Tonic_	µ_BVP_^δ^, min_BVP_^δ^, max_BVP_^δ^
Average RR intervals	—	—	—	µ_RR_
RMSSD^d^ RR interval differences	—	—	—	RMSSD_RR_
Number of peaks	—	—	#_Phasic_	—
Average peak amplitude and response time	—	—	µ_Phasic_^Amp^, µ_Phasic_^t^	—
Power in ULF^e^, LF^f^, HF^g^, and UHF^h^ components	—	—	f_sig_^x^ x ∈ {ULF, LF, HF, UHF} sig ∈ {EDA, Phasic}	—
Mean (SD) of real and imaginary frequency component	—	—	—	RE (µ_BVP_^f^), RE (σ_BVP_^f^), IM (µ_BVP_^f^), IM (σ_BVP_^f^)
Median of real and imaginary frequency component	—	—	—	RE (p^50^_BVP,f_), IM (p^50^_BVP,f_)
IQR of real and imaginary frequency component	—	—	—	RE (p^75^_BVP,f_-p^25^_BVP,f_), IM (p^75^_BVP,f_ -p^25^_BVP,f_)
Minimum and maximum of real and imaginary frequency component	—	—	—	RE (min_BVP_^f^), RE (max_BVP_^f^), IM (min_BVP_^f^), IM (max_BVP_^f^)
Sum of real and imaginary frequencies	—	—	—	RE (∑_BVP_^f^), IM (∑_BVP_^f^)

^a^EDA: electrodermal activity.

^b^BVP: blood volume pulse.

^c^Not applicable.

^d^RMSSD: root mean of successive square differences.

^e^ULF: ultralow frequency.

^f^LF: low frequency.

^g^HF: high frequency.

^h^UHF: ultrahigh frequency.

#### Machine Learning Modeling

To address the feasibility of predicting OCD events from in-the-wild recordings from a machine learning point of view, we make a principal component analysis decomposition of the extracted features from recordings obtained from different known settings. We compared recordings that precede tagged OCD events in the wild during clinical ERP sessions of patients with OCD, the resting phase of patients with OCD, and the physical activity of the control group.

We will consider the prediction of OCD events a binary classification problem. Each time window is considered an independent observation and will be labeled according to the window strategy outlined above. We apply a 2-layer cross-validation strategy with each layer being a random 10-fold cross-validation.

**Figure 3:**
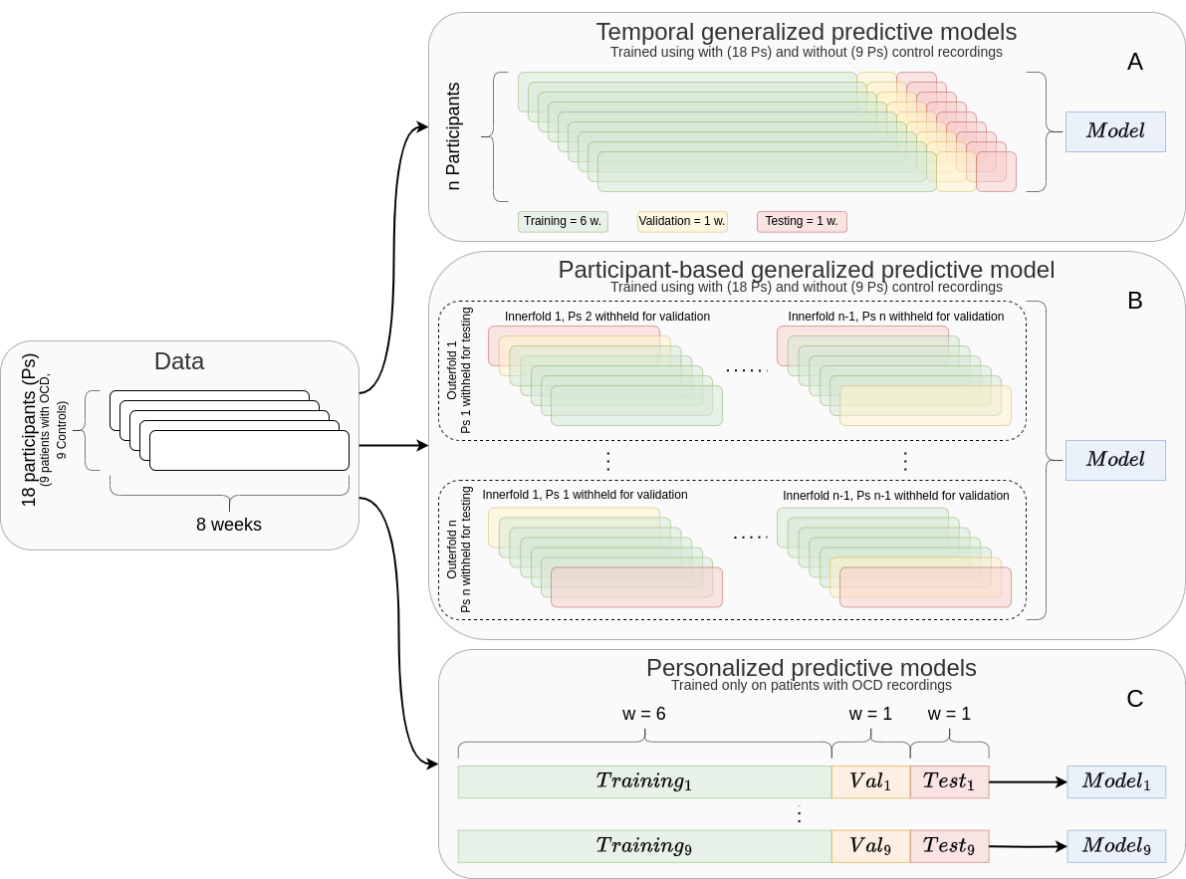
Design of the cross-validation strategies. Temporal generalized predictive models (A) and personalized predictive models (C) use a leave-last-week-out strategy, while participant-based generalized predictive model (B) uses a leave-one-subject-out strategy. OCD: obsessive-compulsive disorder.

We then consider 2 subproblems to study the generalization capabilities of OCD event prediction: generalized predictive models and personalized predictive models as shown in Figure 3. For each subproblem, we apply a 2-layer cross-validation strategy designed to evaluate the generalization across participants and time, respectively. The outer loop estimates model performance by the accuracy, *F*_1_-score, and area under the receiver operating characteristic. The inner loop selects the model and hyperparameters that perform best according to accuracy among logistic regression, random forest, feed-forward neural networks (NNs), and mixed-effect random forest [34].

#### Generalized Predictive Models

In generalized predictive models, we will train models to predict OCD events across patients and for new time periods. We compare the performance of models trained on data solely consisting of patients with OCD and models with data from both patients with OCD and controls.

In participant-based generalized models, we apply 2-layer cross-validation where both the inner and outer layers are leave-one-subject-out. That is, the models are trained on all available participants, except 1 participant who is withheld for testing purposes.

In temporal generalized models, the model is trained and tested on recordings from all participants. The recordings of each participant are divided into 3 data sets for training, validation, and testing. The test data consists of the last week of recording from all participants, and the validation set consists of the recordings from week 7 from all participants.

#### Personalized Predictive Models

Personalized predictive models are trained using the same schema as the temporal generalized models except a different model will be trained for each participant. That is, models are trained and tested on recordings from the same participant. For each participant, the type of model and hyperparameter values are selected using the training and validation data, and model performance is evaluated using the test set.

#### Exploring Deep Learning Models

Once a baseline model based on feature extraction within sampled windows is established, as described above, we will explore the feasibility of deep learning models as a secondary study. In particular, we will consider recurrent NNs [35] or 1D convolutional NNs [36,37]. Feng et al [35] use a combination of 1D convolutions and long short-term memory as feature extractors for stress detection on the WESAD [17] data set, improving the performance over traditional machine learning methods. Chen et al [38] use 1D convolution of 3 physiological signals for automatic emotion recognition. Yu and Sano [37] propose an AutoEncoder-based semisupervised method for stress detection using physiological signals and self-reported stress tags. A benefit of long short-term memories and 1D convolutions is that they can be applied to the recorded time series with little need for preprocessing. We will use the same validation strategies as above. Considering data are limited, we consider this an exploratory proof-of-concept study, and we do not expect results from this study to directly generalize to other studies.

## Results

Participants were included between December 2021 and December 2022. Participants included 10 female and 8 male participants with an even sex distribution between groups. Patients were between the ages of 10 and 16 years, and adolescents without a psychiatric diagnosis were between the ages of 8 and 16 years. Most patients (8/9, 89%) had moderate (CY-BOCS total score between 14 and 24 [39]) to moderate-severe OCD (CY-BOCS total score of 25-30 [39]), except for 1 patient with mild OCD (CY-BOCS total score below 14 [39]).

## Discussion

### Principal Findings

Here, we present a predefined plan for how data will be preprocessed, analyzed, and presented in the publication. If the artificial intelligence tools show promising results, this preregistered analysis plan helps reduce interpretation bias.

We will investigate the predictive generalization of models to newly recorded data and new individuals. Good generalization to new data is required for real-world applications where models are trained on historical data and then expected to perform well on new data recorded in real time. Similarly, if models are found to generalize well to new unseen individuals, they can be applied immediately to new patients without the need for recording data for fine-tuning. This would significantly expedite the application of models on new patients.

The relatively small number of participants In this study could raise issues regarding the generalization of the trained models, especially considering that the data distribution between participants is expected to be very imbalanced. To assess how much data are needed to train predictive models with acceptable performance, we will include a power analysis that compares models trained on different amounts of the available data and estimate the required number of observations to achieve a given performance level using the inverse power law.

As physiological signals may differ heavily from person to person, there is a risk that models trained using multiple people will perform worse than models trained using only 1 person. We assess this issue by comparing the predictive performance of models personalized for each participant and generalized models trained using multiple participants. If personalized models are found to be superior to generalized models, a natural following question is how much labeled data are needed to build such a personalized model and predict OCD cases. Ideally, the data collection period should be as short as possible. For this purpose, it could be interesting to study whether recordings from clinical sessions are sufficient to fine-tune models previously trained on data from a larger corpus.

Models trained solely on patients with OCD are prone to false positives and risk predicting OCD events in individuals without OCD. In future work, we will assess this risk by comparing predictive differences between models trained solely on data recorded from known patients with OCD and models trained using combined patient and control data.

Physiological signals recorded from in-the-wild wearable devices inherently contain high levels of noise and artifacts from the environment, motion, or reflection or absorption properties of different skin tones. This is discussed in van Lier et al [40], using a standardized assessment of the Empatica E4 EDA and BVP measurements compared with established clinical reference devices on multiple levels. Features extracted from the EDA signal using Empatica E4 are found to have significant nonsystematic differences from the same features using the reference device. However, they are still useful in detecting stressful events expected to substantially increase skin conductivity. Moreover, van Lier et al [40] speculate that the “cool-down” period following a stressful event is longer using the E4 than the reference device. This might be problematic for OCD detection as periods following an OCD event could have inflated feature values and be falsely classified as positives. This highlights the need for our proposed block of 5 minutes following each tagged event. For the BVP signal, van Lier et al [40] found a good agreement with the reference device for features related to the heart rate and root mean of successive square differences. However, they also conclude that the E4 has too much data loss due to noise to be useful for prediction of stressful events but argue that the events in their study might not induce enough stress or be of too short duration. The experiments of van Lier et al [40] were conducted in a stationary clinical setting. As such, the issues of data loss must be expected to only increase using in-the-wild data. To combat this issue, we apply the previously explained segmentation strategy to find low-noise segments in the BVP that are suitable for feature extraction.

In this initial study, we discard the parent recording and limit our data to the youth recordings. It is important to understand the relationship between the child tags and the child signals, and we wish to keep the analysis as simple as possible to assess the feasibility. However, in future work, we wish to incorporate data from the parents as well. Children often seek help or reassurance from their parents, which might increase the stress levels of the parent. Thus, we would expect some degree of synchrony between the physiological signals between child and parent. How well the patients cope with their stress levels is expected to be affected by the extent to which the parent accommodates or refuses to accommodate the patient’s symptoms. Refusal to accommodate symptoms or overly accommodating behavior can both increase the stress of the parent at the expense of the patient’s treatment. Additionally, parents may notice stress triggers before the child and attempt to avoid the trigger. Such events can increase the stress of the parent but not the patient. Thus, physiological signals from the parent could be used for parent-focused interventions to ensure the proper supportive treatment. Additionally, the parent tags and signals may also provide insight into how their children’s OCD affects the parents’ peripheral autonomic nervous system responses. This is interesting independently of the children’s signals.

### Potential Clinical Implications

Automated detection of OCD events in the wild may help clinicians assess and monitor the clinical severity of patients waiting for or in treatment. Monitoring distress during ERP and in between sessions allows for data-informed therapy. Automatic feedback will help to increase the patient’s awareness of physiological reactions and the connection between thoughts, feelings, sensations, and actions—a goal of cognitive behavioral therapy [41]. Furthermore, automated detection of OCD events during the build-up phase may serve as a tool for individually tailored ecological momentary interventions, which use upcoming stress events to prompt just-in-time interventions to prevent maladaptive behavior and promote adaptive coping strategies [42]. However, this poses significant requirements to the precision of the predictive models as this function itself could evolve into a source of stress for some patients. Additionally, if the signal characteristics indicating an OCD event are not unique to OCD, it could contribute to an overexaggeration of OCD symptoms if not implemented carefully. This would give clinicians a wrong assessment of the severity of patients and could result in wrong treatment plans. However, comparison with other conditions is outside the scope of this initial feasibility study and left for further research.

### Limitations

In this initial study, we implement a simple windowing approach. We do not have knowledge of whether OCD events were tagged leading up to, during, or after a distress event. For this reason, we consider fairly large time windows to ensure a complete event is captured within each window. Larger time windows were found to increase performance in stress detection in a previous study [16]. The inherent risk associated with large windows is the reduction of the signal-to-noise ratio and the potential overlap of multiple events. Yet, others found that, overall, the window size had little effect on the task of detecting negative emotions using physiological signals in the wild [43]. However, window length was found to affect personalized predictive models differently. As OCD symptoms often differ from patient to patient, we similarly expect that window length will affect participants differently. Therefore, adaptive windowing based on each participant for personalized models might give some knowledge of whether this is an appropriate course to pursue. However, considering the small number of patients included in this initial study, we do not expect conclusive evidence regarding the generalizability of window size. Therefore, a detailed discussion of the relation between window length and symptom type is outside the scope of this study. In future work, we might consider comparing different window lengths for each patient, similar to Gjoreski et al [16].

Event tagging comes with inherent uncertainty. Patients might forget to tag events completely or tag events minutes later after remembering. Moreover, tags might happen by accident or the same event might be tagged multiple times. These issues might lead to falsely labeled positive and negative cases, thereby negatively impacting model training, evaluation, and quality. To circumvent this, Gjoreski et al [16] recommend also labeling windows adjacent to tagged events as positive cases. They argued that an anticipated stressful event might cause physiological arousal leading up to the event as well. However, as OCD events are entirely unplanned, this phenomenon is not expected in our data. On the contrary, falsely labeling windows earlier than the OCD event as positive cases would expectedly negatively impact model precision. In some cases, patients reported that they accidentally tagged events during data collection but could not report the exact time, allowing us to filter such wrong labels. However, these reports in combination with our practical feasibility study might give some insight into the extent of the false labels and allow us to gauge the impact on the model assessment. In the end, a ground truth is required for model training and the best source for internal private processes, such as obsessions or the discomfort caused by obsessions or compulsions, is the person experiencing them. Even if this is sensitive to the problem of human error.

The simple windowing approach ignores the temporal dependence between observations, which may cause false detection of windows in proximity to a labeled OCD event. Future work will investigate a time series–based approach to detect events in the biosignal time series. This could potentially be paired with some of the deep learning methods mentioned in the section “Exploring Deep Learning Models.” A time-series approach may also be useful to address the issues related to the timing of the tags described above. Our current windowing approach treats each tag as an independent event. This may be a fair assumption for events separated by a longer period of time than the cool-down period of 5 minutes. However, for events less than 5 minutes apart, this assumption may not be valid. Patients were asked to tag events when they were bothered by their OCD, which can be interpreted in several ways. When an obsessive thought enters the patient’s mind, the patient tags an event and the first obsessive thought triggers another obsessive thought. Should the patient tag a new event or is this still the same event? A potential prospective future work is to investigate how often tags appear and how dependent they are. Each tag could be used to define a new time series describing the probability of an OCD event currently taking place. At the time of a tag, this probability would be one and then slowly taper off during the period before the tag. This tapering would allow for more uncertainty than the binary tags.

Initially, we disregard recordings from the accelerometer built into the E4 wristband. Acceleration is not expected to be an explanatory signal for the prediction of OCD events. However, as previously discussed, explanatory signals such as EDA and BVP are expected to experience motion artifacts. In previous works, accelerometer data have been used to exclude periods of high movement (see Taylor et al [44], Sarker et al [45], and Hovsepian et al [46]). Initially, we were not interested in excluding periods of high movement as we wish to assess the predictive performance of OCD events during periods of high movement. We intend to study this qualitatively using physical activity and ERP sessions in the clinical setting. Thierfelder et al [2] found that general movement energy increases with higher stress levels; however, that does not mean there is a causality. Thierfelder et al [2] also argue that some compulsive tasks may result in unique frequency spectra. However, this is not common to all compulsive tasks and requires the need for an accelerometer on both hands to look for asymmetric movements. If motion artifacts are found to resemble the changes in physiological signals due to OCD events, we suspect this will result in an increase in type 1 errors (false positives). Gjoreski et al [16] found that an activity context obtained from accelerometer data may reduce the number of false positives. In future work, we will use the laboratory recordings to obtain an understanding of how physiological signals (eg, EDA and BVP) are affected by physical motion compared to OCD events. If these studies find that motion artifacts resemble OCD events, we will aim for an end-to-end solution that explicitly includes the data from the accelerometer in the modeling to reduce the number of false positives.

### Conclusions

In this work, we have discussed the data acquisition and planned analysis for a feasibility study of the prediction of OCD events in the wild using physiological signals recorded from smart wristbands. The current predefined analysis plan will help limit bias for reported results in future publications. We have discussed in detail the potential issues and sources of uncertainty as well as how we plan to address these issues, such as interparticipant and temporal differences, the window length used for feature extraction, event labeling, noisy recordings, and movement-based artifacts. Additionally, we discuss ideas for future work which will improve the understanding of how these limitations affect the problem of predicting OCD events in the wild. If the obtained results from this analysis plan are promising, we will be a step closer to automated detection of OCD events outside of clinical experiments. This is an important tool for the assessment and treatment of OCD in youth.

## Abbreviations

BVP: blood volume pulse
CY-BOCS: Children’s Yale-Brown Obsessive Compulsive Scale
EDA: electrodermal activity
ERP: exposure and response prevention
HRV: heart rate variability
IBI: interbeat interval
NN: neural network
OCD: obsessive-compulsive disorder
PPG: photoplethysmography

## Data Availability

The data sets generated and analyzed during this study are not publicly available due to the sensitive nature of the data and General Data Protection Regulation (GDPR) concerns but are available from the corresponding author on reasonable request.
